# Development of fibrocartilage layers in the anterior cruciate ligament insertion in rabbits

**DOI:** 10.1186/s12891-019-2614-0

**Published:** 2019-05-18

**Authors:** Hirotaka Mutsuzaki, Hiromi Nakajima, Maika Someji, Masataka Sakane

**Affiliations:** 1 Department of Orthopaedic Surgery, Ibaraki Prefectural University of Health Sciences, 4669-2 Ami, Inashiki-gun, Ibaraki, 300-0394 Japan; 2 Department of Agriculture, Ibaraki University, 3-21-1 Chuo, Ami, Ibaraki, 300-0393 Japan; 3 Department of Orthopaedic Surgery, Tsukuba Gakuen Hospital, 2573-1 Kamiyokoba, Tsukuba, Ibaraki, 305-0854 Japan

**Keywords:** Development, Anterior cruciate ligament insertion, Fibrocartilage layers, Chondrocyte, Glycosaminoglycan

## Abstract

**Background:**

A detailed evaluation focusing on the fibrocartilage layers in the anterior cruciate ligament (ACL) insertion is necessary to consider regeneration of the insertion. This study examined the development of the fibrocartilage layers in the ACL tibial insertion in rabbits by quantitative morphometric evaluations based on histological and immunohistochemical analyses.

**Methods:**

Male Japanese white rabbits were used because of their history of use for histomorphometric analyses of the ACL insertion and to eliminate the influence of female hormones on the ACL. Six animals were euthanized at each age (1 day and 1, 2, 4, 6, 8, 12, and 24 weeks); in total, 48 animals were used. Proliferation rate, apoptosis rate, Sox9-positive rate, and chondrocyte number were evaluated. Safranin O-stained glycosaminoglycan (GAG) areas, tidemark length, ACL insertion width, and ACL length were also evaluated. All parameters were compared with those at age 24 weeks of age.

**Results:**

High levels of chondrocyte proliferation and Sox9 expression continued until 4 and 8 weeks of age, respectively, and then gradually decreased. Chondrocyte apoptosis increased up to 8 weeks. The chondrocyte number, ACL insertion width, ACL length, safranin O-stained GAG areas, and tidemark length gradually increased up to 12 weeks.

**Conclusion:**

Chondrocytes that displayed chondrocyte proliferation and Sox9 expression increased until 12 weeks of age, in accordance with development of the ACL length and its insertion width. The GAG production and tidemark length also increased until 12 weeks of age. The development of fibrocartilage layers in the ACL insertion was complete at 12 weeks of age.

**Electronic supplementary material:**

The online version of this article (10.1186/s12891-019-2614-0) contains supplementary material, which is available to authorized users.

## Background

The anterior cruciate ligament (ACL) insertions at the femur and the tibia have four transitional tissue layers: ligament, unmineralized fibrocartilage, mineralized fibrocartilage, and bone (direct-type insertion) [1]. The various degrees of stiffness of these layers reduce the stress concentration at the insertion site [1]. However, only fibrous tissue that was mechanically inferior was noted between the grafted tendon–bone interface after ACL reconstruction using a soft tissue graft (indirect-type insertion) [2–4]. On the other hand, qualitative evaluations revealed an anatomical difference in the ACL insertion structure during growth over time [5, 6]. We consider that optimal treatment at the ACL insertion involves anatomical imitation and/or regeneration of the normal structure. Moreover, specific treatments that approach the normal structure of the ACL insertion at each age are necessary. Therefore, an understanding of the formation process and anatomical structural differences in growth of the fibrocartilage layers (unmineralized and mineralized fibrocartilage) in the ACL insertion is necessary when considering the most appropriate treatment strategy based on age and the development of new treatment methods for regeneration of the tendon–bone interface.

We focused on the fibrocartilage layers as a load transmitter because glycosaminoglycans (GAGs) in the fibrocartilage layers provide tissue elasticity [7]. The GAGs provide resistance to tensile, shear, and compressive stresses and are thus important for load transmission [1, 7]. In our previous reports, we showed that mechanical unloading and knee immobilization increased chondrocyte apoptosis, decreased chondrocyte proliferation, and decreased the GAGs in the fibrocartilage layers in the patellar tendon insertion and ACL insertion in rabbits [8, 9]. Conversely, over load via an ACL partial tear and gradual elongation using external fixation decreased chondrocyte apoptosis, increased chondrocyte proliferation, and increased the GAGs in the fibrocartilage layers in the patellar tendon insertion and ACL insertion in rabbits [10, 11]. Therefore, we consider that chondrocyte apoptosis, chondrocyte proliferation, and GAGs can effectively reflect differences in the mechanical environment at the insertion site. Moreover, sex-determining region Y box 9 (Sox9) directly regulates the type-II collagen gene and is a master regulator of chondrogenesis by promoting proliferation and differentiation of mesenchymal stem cells into chondrocytes [12–14].

We considered that a more detailed quantitative evaluation using these parameters (i.e., chondrocyte apoptosis, chondrocyte proliferation, Sox9, and GAGs) when focusing on the fibrocartilage layers in the ACL insertion is crucial to understanding the formation process and the anatomical structural differences of the fibrocartilage layers during growth and healing of the ACL insertion.

The purpose of this study was to evaluate the development of the fibrocartilage layers in the ACL tibial insertion in rabbits by quantitative morphometry evaluations based on histological and immunohistochemical analyses.

## Methods

### Animal preparation

This laboratory-based animal study used a judgement sampling technique. Forty-eight male Japanese white rabbits were used because of their history of use for histomorphometric analyses of the ACL insertion and to eliminate the influence of female hormones on the ACL [8–11, 15]. Because the skeletal growth of rabbits is complete at 6 months [16], we set the evaluation period at 24 weeks of age. These rabbits were purchased from Japan SLC, Inc. (Hamamatsu, Japan) and Hamada farm (Miho, Japan). The rabbits were maintained in accordance with the guidelines of the Ethical Committee of Ibaraki Prefectural University of Health Sciences and Ibaraki University, and the National Institutes of Health Guidelines for the Care and Use of Laboratory Animals (NIH Pub. No. 86–23 Rev. 1985). Six animals at each age (1 day, 1, 2, 4, 6, 8, 12, and 24 weeks) were euthanized by over dose intravenous barbiturate injection (200 mg/kg, Somnopentyl®, Kyoritsu Seiyaku Corporation, Tokyo, Japan). Because this study was an evaluation of the development of fibrocartilage layers in ACL tibial insertions, it could not be considered an in vitro experiment. The animal species to be studied was determined based on previous reports [8–11]. Moreover, it is considered difficult to prepare tissue specimens using animals smaller than rabbits. Therefore, we chose rabbits for this study.

### Histomorphological analysis

**Fig. 1:**
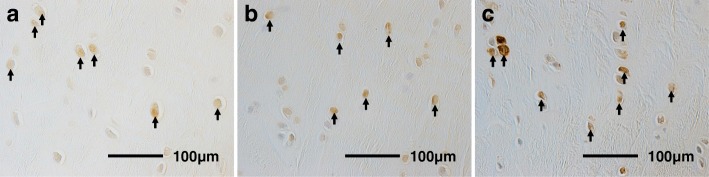
Stained histological sections. **a** PCNA staining (400×). PCNA-positive chondrocytes are brown (arrows). **b** TUNEL staining (400×). TUNEL-positive chondrocytes are brown (arrows). Sox9 staining (400×). **c** Sox9-positive chondrocytes are brown (arrows). PCNA, proliferating cell nuclear antigen; TUNEL: terminal deoxynucleotidyl transferase-mediated deoxyuridine triphosphate-biotin nick-end labeling; Sox9: sex-determining region Y box 9

Knees from the animals were fixed in 10% neutral-buffered formalin for 1 week. After fixation, the specimens from rabbits 2–24 weeks of age were decalcified in 10% ethylenediaminetetraacetic acid (pH 7.4) for 7–12 weeks and then embedded in paraffin. The specimens from rabbits 1 day and 1 weeks of age could be sliced without decalcification. The specimens were sliced at 5-μm thickness in the center of the ACL tibial insertion site. The slices were stained with hematoxylin-eosin, and safranin O to assess the histomorphology and GAG contents [8–11]. We also used proliferating cell nuclear antigen (PCNA) staining to detect proliferating cells [8–11] (Fig. 1a), terminal deoxynucleotidyl transferase-mediated deoxyuridine triphosphate-biotin nick-end labeling (TUNEL) staining to detect apoptotic cells [8–11] (Fig. 1b), and Sox9 staining to evaluate the developmental differentiation of chondrocytes (Fig. 1c).

PCNA immunostaining was carried out with a Histofine® SAB-PO(M) Kit (Nichirei Biosciences Inc., Tokyo, Japan) according to the manufacturers’ instructions. Briefly, sections were deparaffinized, rinsed in phosphate-buffered saline (PBS) for 5 min, and immersed in 3% hydrogen peroxide (H_2_O_2_) in methanol for 10 min to block endogenous peroxidase activity. After rinsing in PBS for 5 min, the sections were blocked in 10% normal rabbit serum at 25 °C for 10 min, and incubated with an anti-PCNA monoclonal antibody (PC-10; Code No. M0879; Dako, Glostrup, Denmark) at 1:100 dilution for 12 h at 4 °C. Antibody Diluent (Code No. S0809; Dako) was used instead of a primary antibody for the negative controls [8–11].

TUNEL staining was performed using an Apoptag® Plus Peroxidase In Situ Apoptosis Detection Kit (Merck Millipore, Billerica, MA, USA) according to the manufacturers’ instructions. TUNEL-positive nuclei of chondrocytes were stained dark brown, and TUNEL-negative nuclei were stained blue [8–11].

Sox9 immunohistochemical staining was performed with a Histofine® SAB-PO (R) Kit (Nichirei Biosciences Inc.) and a Rabbit-To-Rabbit Blocking Reagent (ScyTek Laboratories Inc., Logan, UT, USA) according to the manufacturers’ instructions. Deparaffinized sections were rinsed twice with PBS for 3 min each and immersed in 3% H_2_O_2_ in methanol for 10 min to block endogenous peroxidase activity. After three rinses in PBS for 5 min each, the sections were blocked with 10% normal goat serum at room temperature for 10 min, incubated with a Rabbit-To-Rabbit Blocking Reagent at room temperature for 30 min, and washed four times in PBS for 5 min each. The sections were then incubated with an anti-Sox9 rabbit polyclonal antibody (Bioworld Technology Inc., Louis Park, MN, USA) at 1:100 dilution for 24 h at 4 °C. The immunoreaction product was developed in diaminobenzidine, and the sections were counterstained with Mayer’s hematoxylin for 30 s. Sox9-positive nuclei were stained dark brown and Sox9-negative nuclei were stained blue.

**Fig. 2:**
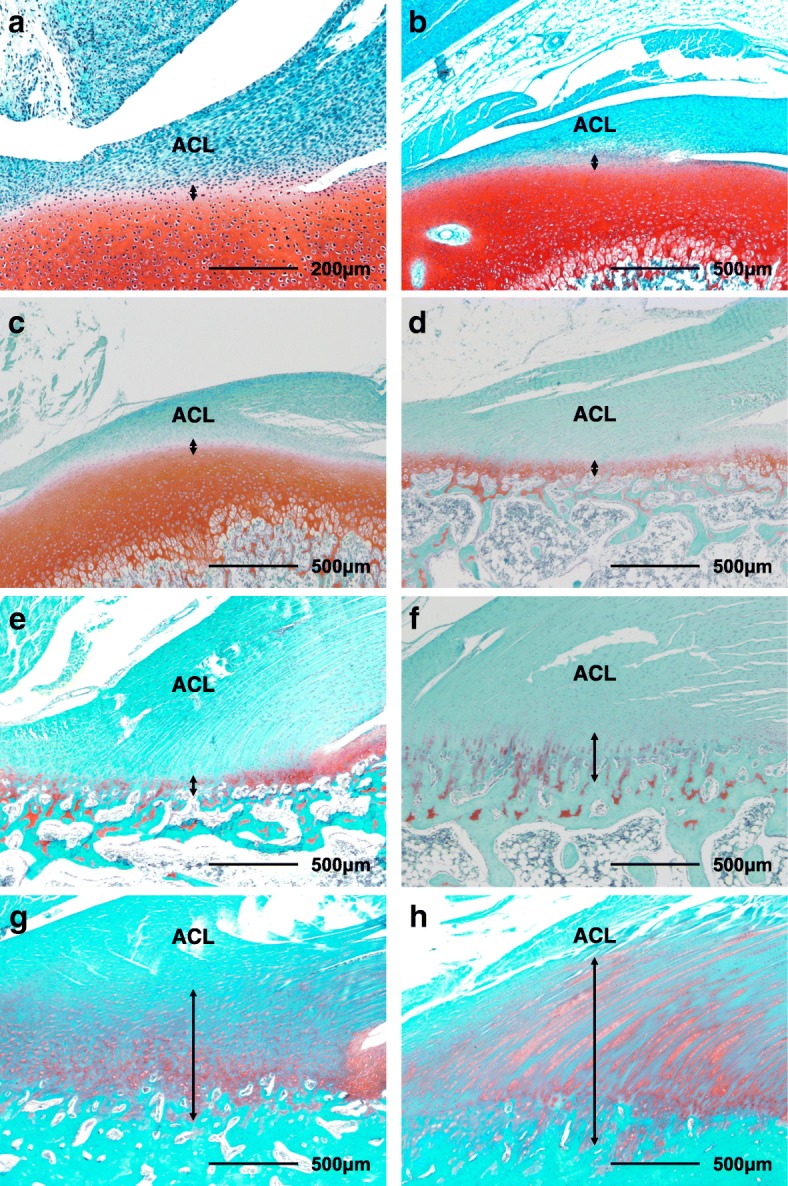
Representative histological sections of anterior cruciate ligament tibial insertions at each age stained with safranin O. The interfacial fibrocartilage layers (black arrows) were evaluated. Images for each rabbit age are shown: (**a**) 1 day (100×). (**b**) 1 week (40×). (**c**) 2 weeks (40×). (**d**) 4 weeks (40×). (**e**) 6 weeks (40×). (**f**) 8 weeks (40×). (**g**) 12 weeks (40×). (h) 24 weeks (40×). ACL, anterior cruciate ligament

Histomorphometric analyses were performed using similar methods to those in our previous study [8–11]. The sections were examined using a BX-51 light microscope (Olympus Optical Co. Ltd., Tokyo, Japan). The GAG areas stained red by safranin O were evaluated in the fibrocartilage layers in the ACL tibial insertion (Fig. 2). In the specimens from rabbits 1 day and 1 and 2 weeks of age, we defined the fibrocartilage layers in the ACL tibial insertion as those having lower-density staining of cartilaginous tissue than articular cartilage by safranin O with round cells, and between the ligament and hyaline cartilage area continuous with articular cartilage. In the specimens from rabbits ≥4 weeks of age, we defined the fibrocartilage layers in the ACL tibial insertion as the cartilaginous tissue with round cells between the ligament and bone [9, 10]. The length of the tidemark in the ACL tibial insertion was measured as the sum-total length that stained with hematoxylin-eosin. The histological ACL length was defined as the distance between the anterior attachment of the femur and the posterior attachment of the tibia [6]. The width of the ACL tibial insertion was defined as the anterior-to-posterior distance of the ACL tibial attachment. Mac Scope software (Mitani Co., Fukii, Japan) was used to determine the total numbers of chondrocytes and the numbers of TUNEL-positive, PCNA-positive, and Sox9-positive chondrocytes in the safranin O-stained GAG areas in the fibrocartilage layers in the ACL tibial insertion. Each red-stained GAG area and tidemark length was divided by the width of the ACL insertion to define the average thickness of the red-stained GAG areas and the percentage of the tidemark length relative to the ACL insertion width, respectively. The TUNEL-, PCNA-, and Sox9-positive rates were calculated based on the total numbers of chondrocytes in the safranin O-stained GAG areas in the fibrocartilage layers.

### Statistical analysis

For each parameter, normality of the data was tested using the Shapiro-Wilk normality test. The time-dependent histological changes were evaluated by one-way analysis of variance (ANOVA) when the assumption of normality of all variables for each parameter was accepted. Factors determined to show significant differences by ANOVA were further evaluated by Dunnett’s test. When the assumption of normality failed for all variables in each parameter, the Kruskal-Wallis test and Bonferroni adjustment technique were applied. All parameters were compared with those at 24 weeks. The level of significance was set at 5%. All analyses were performed with IBM SPSS Statistics version 24.0 (IBM Corp., Armonk, NY, USA).

According to a previous study [9], a power calculation was performed with a confidence level of 95% (α = 0.05) and power (1 – β) of 80% using the POWER Procedure in SAS software (SAS Institute, Cary, NC, USA). Calculation of the smallest sample size that produced a significant difference yielded an estimated sample size of five to six specimens per age group. We enrolled six specimens per age group to reduce the number of animals used.

## Results

All 48 rabbits were used for the evaluations. The Shapiro-Wilk test showed that the numbers of chondrocytes, thicknesses of safranin O-stained GAG areas, and width of the ACL insertion were normality distributed. The data for the other parameters were not normality distributed.

### Chondrocyte proliferation rate

**Fig. 3:**
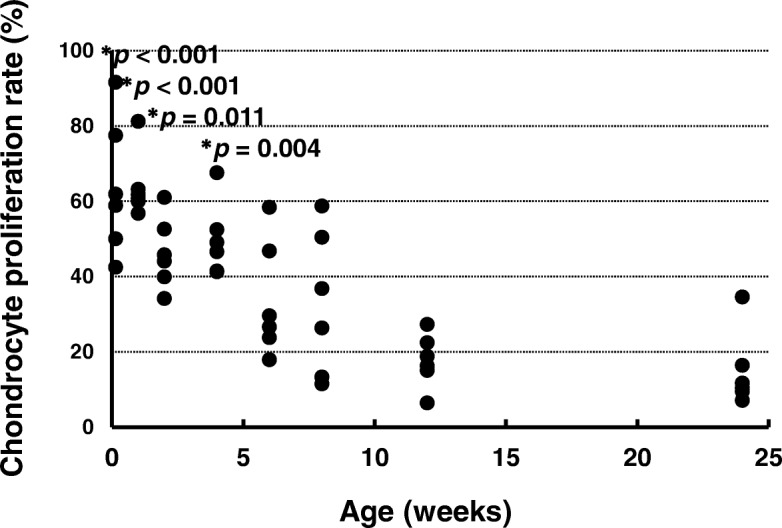
Chondrocyte proliferation rates. *p < 0.05 versus age 24 weeks (*n* = 6)

The chondrocyte proliferation rate was determined by the numbers of PCNA-positive chondrocytes (Fig. 3). The chondrocyte proliferation rates at ages 1 day (*p* < 0.001), 1 week (*p* < 0.001), 2 weeks (*p* = 0.011), and 4 weeks (*p* = 0.004) were significantly higher than that at age 24 weeks. There were no significant differences between the chondrocyte proliferation rates at ages 6 weeks (*p* = 0.103), 8 weeks (*p* = 0.138), and 12 weeks (*p* = 0.757) and that at age 24 weeks (effect size: *r* = 4.694, power: 1.000).

### Chondrocyte apoptosis rate

**Fig. 4:**
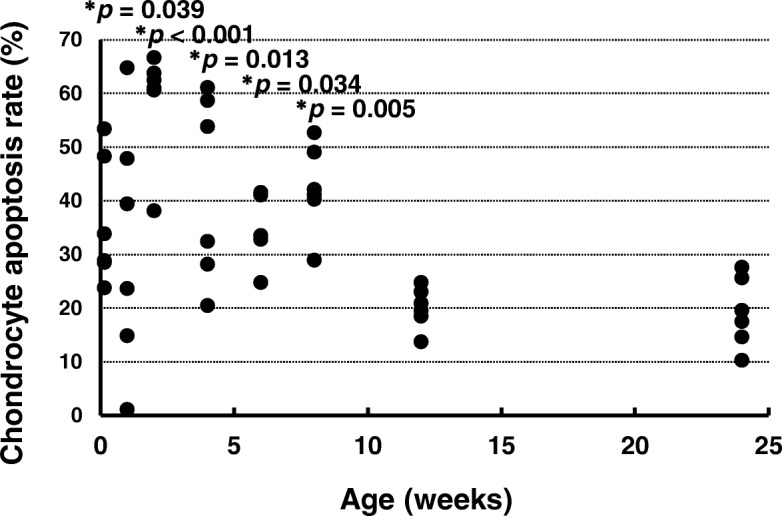
Chondrocyte apoptosis rates. *p < 0.05 versus age 24 weeks (*n* = 6)

The chondrocyte apoptosis rate was determined by the numbers of TUNEL-positive chondrocytes (Fig. 4). The chondrocyte apoptosis rates at ages 1 day (*p* = 0.039), 2 weeks (*p* < 0.001), 4 weeks (*p* = 0.013), 6 weeks (*p* = 0.034), and 8 weeks (*p* = 0.005) were significantly higher than that at age 24 weeks. There were no significant differences between the chondrocyte apoptosis rates at ages 1 week (*p* = 0.132) and 12 weeks (*p* = 0.984) and that at age 24 weeks (effect size: *r* = 3.754, power: 0.980).

### Developmental differentiation of chondrocytes

**Fig. 5:**
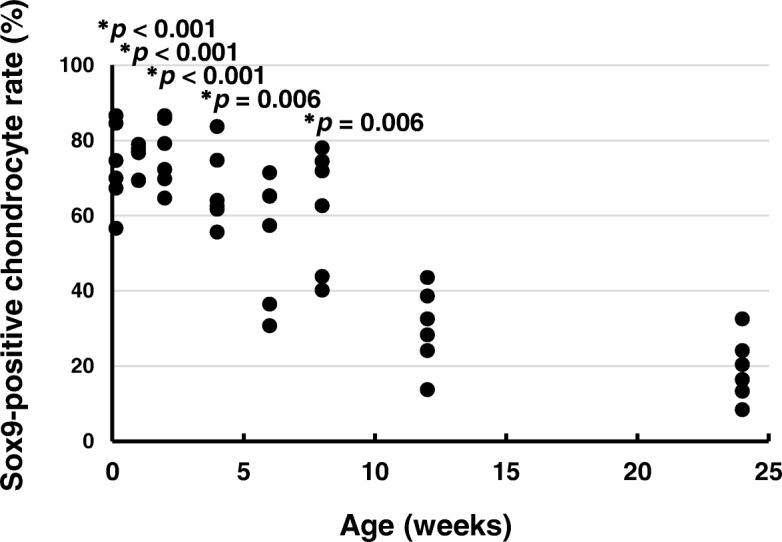
Sox9-positive chondrocyte rates. *p < 0.05 versus age 24 weeks (*n* = 6)

The developmental differentiation of chondrocytes was determined by the numbers of Sox9-positive chondrocytes (Fig. 5). The Sox9-positive chondrocyte rates at ages 1 day (*p* < 0.001), 1 week (*p* < 0.001), 2 weeks (*p* < 0.001), 4 weeks (*p* = 0.006), and 8 weeks (*p* = 0.006) were significantly higher than that at age 24 weeks. There was no significant difference between the Sox9-positive chondrocyte rate at age 6 weeks (*p* = 0.053) and 12 weeks (*p* = 0.578) and that at age 24 weeks (effect size: *η*_*p*_^*2*^ = 4.579, power: 1.000).

### Numbers of chondrocytes in the ACL insertion

**Fig. 6:**
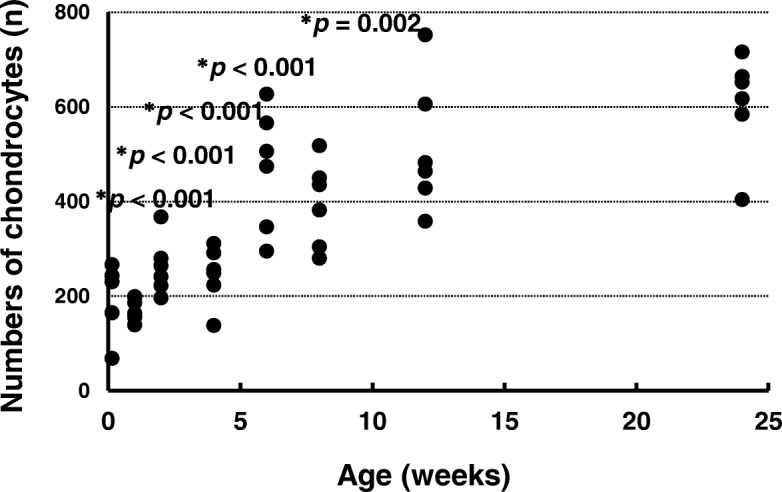
Numbers of chondrocytes in the anterior cruciate ligament tibial insertion. *p < 0.05 versus age 24 weeks (*n* = 6)

The numbers of chondrocytes in the ACL insertion are shown in Fig. 6. The numbers of chondrocytes at ages 1 day (*p* < 0.001), 1 week (*p* < 0.001), 2 weeks (*p* < 0.001), 4 weeks (*p* < 0.001), and 8 weeks (*p* = 0.002) were significantly lower than that at age 24 weeks. There were no significant differences between the number of chondrocytes at ages 6 weeks (*p* = 0.076) and 12 weeks (*p* = 0.390) and that at age 24 weeks (effect size: *η*_*p*_^*2*^ = 0.761, power: 0.991).

### Thickness of safranin O-stained GAG areas

**Fig. 7:**
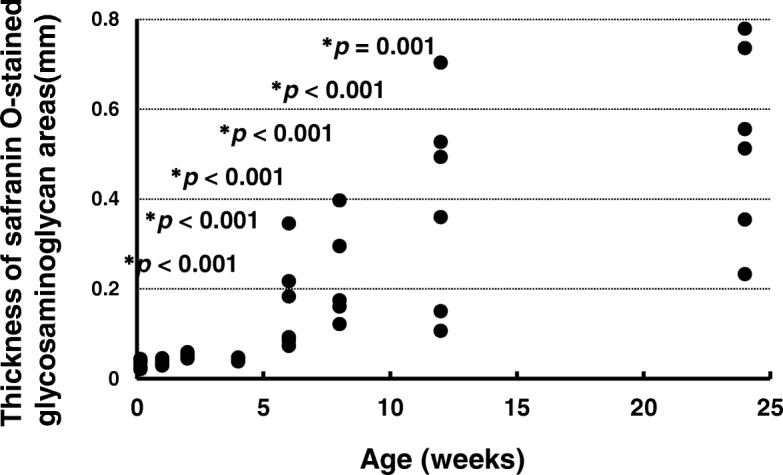
Thicknesses of safranin O-stained glycosaminoglycan areas in the anterior cruciate ligament tibial insertion. *p < 0.05 versus age 24 weeks (*n* = 6)

The thicknesses of the safranin O-stained GAG areas are shown in Fig. 7. The thicknesses at ages 1 day (*p* < 0.001), 1 week (*p* < 0.001), 2 weeks (*p* < 0.001), 4 weeks (*p* < 0.001), 6 weeks (*p* < 0.001), and 8 weeks (*p* = 0.001) were significantly smaller than that at age 24 weeks. There was no significant difference between the thickness at age 12 weeks (*p* = 0.255) and that at age 24 weeks (effect size: *η*_*p*_^*2*^ = 0.711, power: 1.000).

### Percentages of tidemark length relative to ACL insertion width

**Fig. 8:**
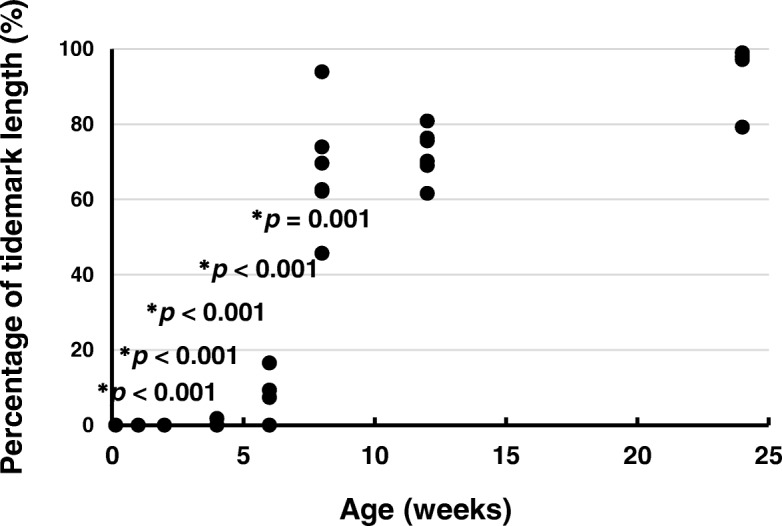
Percentages of tidemark length relative to the anterior cruciate ligament tibial insertion width. *p < 0.05 versus age 24 weeks (*n* = 6)

The percentages of the tidemark length relative to the ACL insertion width are shown in Fig. 8. The percentages at ages 1 day (*p* < 0.001), 1 week (*p* < 0.001), 2 weeks (*p* < 0.001), 4 weeks (*p* < 0.001), and 6 weeks (*p* = 0.001) were significantly lower than that at age 24 weeks. There was no significant difference between the percentages at ages 8 weeks (*p* = 0.208) and 12 weeks (*p* = 0.301) and that at age 24 weeks (effect size: *r* = 6.170, power: 1.000).

### Length of ACL

**Fig. 9:**
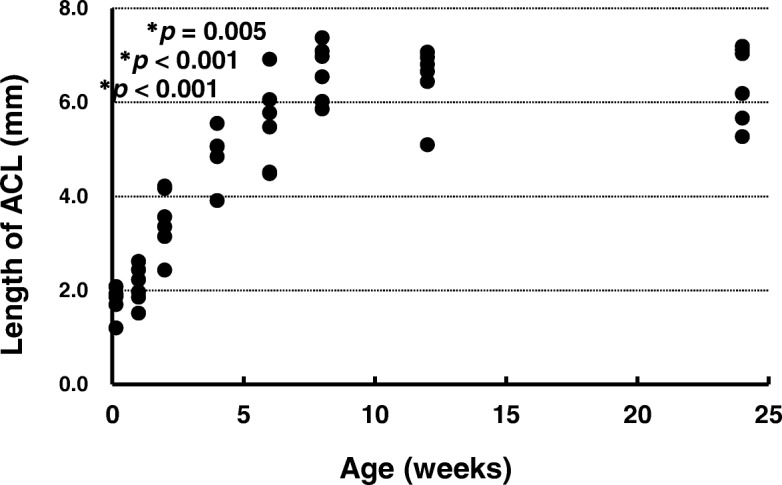
Length of anterior cruciate ligament. *p < 0.05 versus age 24 weeks (*n* = 6). ACL, anterior cruciate ligament

The ACL lengths are shown in Fig. 9. The lengths at ages 1 day (*p* < 0.001), 1 week (*p* < 0.001), and 2 weeks (*p* = 0.005), were significantly smaller than that at age 24 weeks. There were no significant differences between the lengths at ages 4 weeks (*p* = 0.070), 6 weeks (*p* = 0.266), 8 weeks (*p* = 0.853), and 12 weeks (*p* = 0.934) and that at age 24 weeks (effect size: *r* = 5.811, power: 1.000).

### Width of ACL insertion

**Fig. 10:**
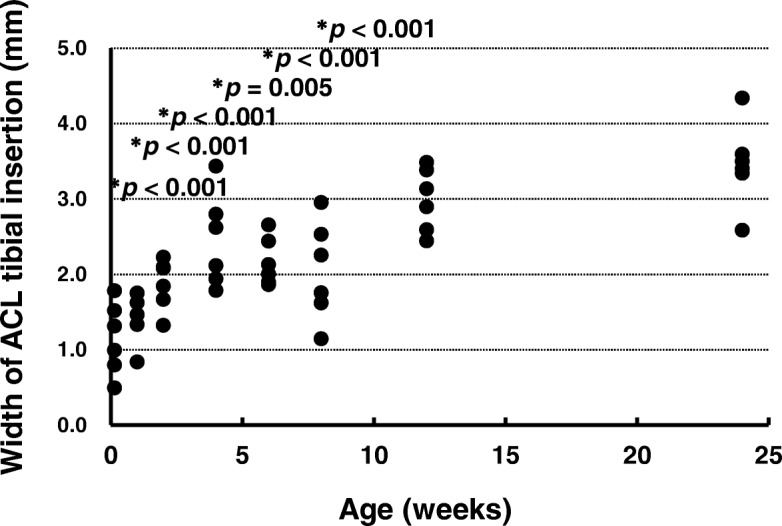
Width of anterior cruciate ligament tibial insertion. *p < 0.05 versus age 24 weeks (*n* = 6). ACL, anterior cruciate ligament

The ACL insertion widths are shown in Fig. 10. The widths at ages 1 day (*p* < 0.001), 1 week (*p* < 0.001), 2 weeks (*p* < 0.001), 4 weeks (*p* = 0.005), 6 weeks (*p* < 0.001), and 8 weeks (*p* < 0.001) were significantly smaller than that at age 24 weeks. There was no significant difference between the width at age 12 weeks (*p* = 0.393) and that at age 24 weeks (effect size: *η*_*p*_^*2*^ = 0.721, power: 1.000).

## Discussion

High levels of chondrocyte proliferation and Sox9 expression in the ACL tibial insertion continued until 4 and 8 weeks of age, respectively, and then gradually decreased. Although the increased chondrocyte apoptosis rates continued until 8 weeks, the chondrocyte numbers gradually increased up to age 12 weeks. The ACL length and insertion width gradually increased until ages 4 and 12 weeks, respectively. The safranin O-stained GAG areas gradually enlarged up to age 12 weeks, and the tidemark gradually widened up to age 8 weeks.

Until 12 weeks of age, the number of chondrocyte in the ACL tibial insertion gradually increased, based on the increased chondrocyte proliferation and Sox9 expression. This result supports previous qualitative findings reported by Nawata et al. [6], who detected that PCNA-stained chondrocytes were detected at the ACL tibial insertion until 1 month of age in rats [6]. In the present study, increased chondrocyte apoptosis was observed until 8 weeks of age. Chondrocyte proliferation may outpace the chondrocyte apoptosis in the growth period. Therefore, the number of chondrocytes gradually increased up to age 12 weeks. Given the similarities with the growth plate, the early stages of ACL insertion development may involve control of chondrocyte proliferation, followed by apoptosis [14].

Until 4 to 12 weeks of age, the ACL length, the ACL tibial insertion width, the GAG production and the tidemark length increased in the ACL tibial insertion. The increase in safranin O-stained GAG areas might have been due to mechanical stresses and an increased total number of chondrocytes. Moreover, the tidemark, which is the mechanical boundary between the unmineralized and mineralized fibrocartilage zones, is important to reduce damage to soft tissues during joint movement [1]. Mechanical stresses may be necessary for formation of the tidemark. In accordance with gait and skeletal growth, the ACL undergoes tensile, shear, and compressive stresses. Such mechanical stresses is important for development of the fibrocartilage layers in the ACL insertion [17, 18]. GAGs, which provide tissue hydration and elasticity in the ligament insertion, also resist mechanical stresses [1]; therefore increased GAG production may be associated with increased mechanical stresses at the ACL insertion site. Gao et al. [19] reported that the medial collateral ligament in the rat was attached to the hyaline cartilage that initially preceded the bone rudiment. The hyaline cartilage was then eroded, but fibrocartilage appeared in the ligament insertion because of metaplasia of the fibroblasts. They suggested that the metaplasia was probably driven by mechanical stimuli associated with movement of the ligament relative to the bone at the insertion site.

The tendon–bone interface is unable to regenerate a direct-type insertion, and instead forms an indirect-type insertion with inferior biomechanical properties that can potentially lead to osteoarthritis [2–4, 20–22]. Based on our results, increased chondrocytes at the tendon–bone interface during the early phase after ACL reconstruction may be important for the formation of the fibrocartilage layers. Previous reports have described good outcomes after administration of growth factors (e.g., bone morphogenetic protein [23], fibroblast growth factor [24], and transforming growth factor-β [25]) and transplantation of multipotent cells and tissues that encourage improved tendon–bone healing with the fibrocartilage layers [26, 27]. Moreover, appropriate mechanical stresses at the tendon–bone interface and the loading period after ACL reconstruction can be important factors leading to maturation and completion of the ACL insertion with the fibrocartilage layers and tidemark. Anatomical structural differences accompanying growth of the fibrocartilage layers in the ACL insertion were also clarified in the present study. Hence, an appropriate treatment strategy based on age could be considered.

The limitations of this study are as follows. Although we performed quantitative analyses up to 6 months of age (i.e., when the skeletal growth of rabbits is complete [16]), evaluations after 6 months may be also necessary when examining the fibrocartilage layer after the growth period. In future studies, mechanical analyses will be necessary to clarify the association with mechanical stresses. Because only the phenotype was investigated in this study, clarification of the complex network of signaling systems and pathways is needed.

## Conclusions

Until the studied rabbits reached 12 weeks of age, chondrocytes that exhibited chondrocyte proliferation and Sox9 expression increased in accordance with the gradual development of the ACL length and insertion width. The GAG production and tidemark length also increased until 12 weeks of age. The development of fibrocartilage layers in the ACL insertion was completed at 12 weeks of age.

## Additional file

Additional file 1: The raw data of this experiment. (DOCX 17 kb)

## Abbreviations

ACL: Anterior cruciate ligament
ANOVA: Analysis of variance
GAG: Glycosaminoglycan
H_2_O_2_: Hydrogen peroxide
PBS: Phosphate-buffered saline
PCNA: Proliferating cell nuclear antigen
Sox9: Sex-determining region Y box 9
TUNEL: Terminal deoxynucleotidyl transferase-mediated deoxyuridine triphosphate-biotin nick-end labeling
