# Supplementary material for: Development of fibrocartilage layers in the anterior cruciate ligament insertion in rabbits
Source: BMC Musculoskelet Disord. 2019 May 18;20:233. doi: 10.1186/s12891-019-2614-0 (PMC6525973; doi:10.1186/s12891-019-2614-0)
Supplement: Supplementary file 1 — The raw data of this experiment. (DOCX 17 kb) [file 12891_2019_2614_MOESM1_ESM.docx]

Chondrocyte proliferation rates.

| 1 day | 1 week | 2 weeks | 4 weeks | 6 weeks | 8 weeks | 12 weeks | 24 weeks |
| --- | --- | --- | --- | --- | --- | --- | --- |
| 77.5 | 63.19018 | 45.75646 | 46.57534 | 17.91045 | 13.29114 | 6.451613 | 9.490334 |
| 91.56627 | 81.20805 | 34.14634 | 41.42395 | 29.5499 | 26.29969 | 15.06849 | 10.3139 |
| 61.8705 | 60.7362 | 60.98901 | 41.21864 | 58.35616 | 36.7893 | 18.76209 | 16.44042 |
| 58.86076 | 60 | 44.05286 | 49.0566 | 26.56766 | 58.69565 | 22.44444 | 7.099698 |
| 42.48705 | 61.76471 | 39.8773 | 52.43056 | 23.78472 | 11.50794 | 16.26734 | 34.59016 |
| 50 | 56.75676 | 52.55474 | 67.53927 | 46.76259 | 50.42493 | 27.27273 | 11.71171 |

Chondrocyte apoptosis rates.

| 1 day | 1 week | 2 weeks | 4 weeks | 6 weeks | 8 weeks | 12 weeks | 24 weeks |
| --- | --- | --- | --- | --- | --- | --- | --- |
| 23.78049 | 47.85276 | 62.5 | 53.81166 | 24.74576 | 28.92857 | 18.45794 | 17.46575 |
| 33.82353 | 64.7482 | 66.66667 | 32.42188 | 33.49282 | 42.10526 | 19.50207 | 10.24096 |
| 53.38346 | 14.83871 | 60.99585 | 20.48193 | 41.51943 | 40.22989 | 20.90517 | 27.60736 |
| 48.26087 | 23.61809 | 63.77551 | 58.69565 | 41.13924 | 52.66667 | 13.68715 | 19.55307 |
| 28.80658 | 39.39394 | 38.14714 | 28.17869 | 32.80632 | 41.09948 | 23.00532 | 25.60778 |
| 28.48485 | 1.075269 | 60.60606 | 61.09325 | 41.04046 | 49.03475 | 24.75248 | 14.60396 |

Sox9-positive chondrocyte rates.

| 1 day | 1 week | 2 weeks | 4 weeks | 6 weeks | 8 weeks | 12 weeks | 24 weeks |
| --- | --- | --- | --- | --- | --- | --- | --- |
| 70 | 77.71739 | 72.28916 | 55.61224 | 30.73593 | 43.79947 | 13.71681 | 20.4 |
| 86.56716 | 77.16049 | 64.67662 | 64.0553 | 57.37977 | 40.1662 | 28.31683 | 13.29305 |
| 74.66063 | 69.32515 | 85.84906 | 62.5 | 65.14886 | 71.86761 | 38.62816 | 16.41104 |
| 84.53608 | 78.94737 | 69.79167 | 61.68831 | 65.28067 | 77.98165 | 43.51396 | 8.370703 |
| 56.65399 | 76.71233 | 86.57407 | 83.67347 | 36.45621 | 62.61128 | 24.12523 | 24.12523 |
| 67.28395 | 69.47791 | 79.14692 | 74.72826 | 71.42857 | 74.45483 | 32.56881 | 32.56881 |

Numbers of chondrocytes in the anterior cruciate ligament tibial insertion.

| 1 day | 1 week | 2 weeks | 4 weeks | 6 weeks | 8 weeks | 12 weeks | 24 weeks |
| --- | --- | --- | --- | --- | --- | --- | --- |
| 164 | 163 | 280 | 223 | 295 | 280 | 428 | 584 |
| 68 | 139 | 222 | 256 | 627 | 304 | 482 | 664 |
| 266 | 155 | 241 | 249 | 566 | 435 | 464 | 652 |
| 230 | 199 | 196 | 138 | 474 | 450 | 358 | 716 |
| 243 | 198 | 367 | 291 | 506 | 382 | 752 | 617 |
| 165 | 186 | 264 | 311 | 346 | 518 | 606 | 404 |

Thicknesses of safranin O-stained glycosaminoglycan areas in the anterior cruciate ligament tibial insertion.

| 1 day | 1 week | 2 weeks | 4 weeks | 6 weeks | 8 weeks | 12 weeks | 24 weeks |
| --- | --- | --- | --- | --- | --- | --- | --- |
| 0.02121 | 0.03435 | 0.04593 | 0.04704 | 0.09229 | 0.29512 | 0.52716 | 0.35397 |
| 0.04403 | 0.02943 | 0.04934 | 0.04295 | 0.34559 | 0.16064 | 0.49359 | 0.73574 |
| 0.04009 | 0.04546 | 0.04518 | 0.03934 | 0.08462 | 0.17463 | 0.15023 | 0.51219 |
| 0.03625 | 0.03906 | 0.04928 | 0.0472 | 0.18331 | 0.39678 | 0.10627 | 0.77916 |
| 0.02485 | 0.03666 | 0.05303 | 0.04427 | 0.21719 | 0.29484 | 0.70351 | 0.55564 |
| 0.02533 | 0.0318 | 0.05918 | 0.03865 | 0.07279 | 0.12172 | 0.35984 | 0.23297 |

Percentages of tidemark length relative to the anterior cruciate ligament tibial insertion width.

| 1 day | 1 week | 2 weeks | 4 weeks | 6 weeks | 8 weeks | 12 weeks | 24 weeks |
| --- | --- | --- | --- | --- | --- | --- | --- |
| 0 | 0 | 0 | 1.776459 | 9.298292 | 45.71707 | 76.32856 | 98.97143 |
| 0 | 0 | 0 | 0 | 7.39891 | 62.63538 | 69.03401 | 97.18044 |
| 0 | 0 | 0 | 0 | 16.56772 | 93.95458 | 75.59609 | 97.1666 |
| 0 | 0 | 0 | 0 | 0 | 69.63962 | 70.17049 | 97.91849 |
| 0 | 0 | 0 | 0 | 0 | 62.14852 | 61.60765 | 79.20594 |
| 0 | 0 | 0 | 0 | 0 | 73.98841 | 80.86497 | 97.96649 |

Length of anterior cruciate ligament.

| 1 day | 1 week | 2 weeks | 4 weeks | 6 weeks | 8 weeks | 12 weeks | 24 weeks |
| --- | --- | --- | --- | --- | --- | --- | --- |
| 1.6975 | 2.22526 | 4.1708 | 5.05561 | 4.4837 | 6.0189 | 6.95874 | 7.03292 |
| 1.92426 | 2.43828 | 4.21723 | 4.8409 | 5.7794 | 5.8575 | 6.81008 | 7.11566 |
| 1.88035 | 1.85117 | 3.35218 | 5.0701 | 5.4757 | 7.0912 | 6.44304 | 5.27139 |
| 2.07731 | 1.51476 | 3.56454 | 3.9113 | 6.0595 | 6.9754 | 5.09841 | 6.18471 |
| 1.86217 | 1.96545 | 3.14255 | 5.54975 | 6.91855 | 7.3752 | 7.06668 | 7.19245 |
| 1.198 | 2.61405 | 2.43122 | 4.84577 | 4.51631 | 6.54161 | 6.65912 | 5.66235 |

Width of anterior cruciate ligament tibial insertion.

| 1 day | 1 week | 2 weeks | 4 weeks | 6 weeks | 8 weeks | 12 weeks | 24 weeks |
| --- | --- | --- | --- | --- | --- | --- | --- |
| 0.99173 | 1.62469 | 1.3218 | 1.7867 | 1.8968 | 2.2543 | 2.89374 | 3.59528 |
| 0.49379 | 0.83615 | 1.66727 | 2.7971 | 2.4409 | 2.5317 | 3.38297 | 4.3404 |
| 1.78293 | 1.74758 | 1.8422 | 2.1155 | 2.0045 | 1.75455 | 2.4426 | 3.49862 |
| 1.31145 | 1.46656 | 2.09437 | 1.9418 | 2.1283 | 1.62162 | 2.59012 | 3.40282 |
| 1.51791 | 1.33464 | 2.08159 | 2.62345 | 1.8638 | 1.14339 | 3.13401 | 3.34004 |
| 0.79526 | 1.7512 | 2.22844 | 3.43474 | 2.65698 | 2.95426 | 3.4845 | 2.58469 |
